# Draft Genome Sequence of *Bacillus thuringiensis* Strain LM1212, Isolated from the Cadaver of an *Oryctes gigas* Larva in Madagascar

**DOI:** 10.1128/genomeA.00516-14

**Published:** 2014-05-29

**Authors:** Guiming Liu, Chao Deng, Lai Song, Qi Peng, Jie Zhang, Didier Lereclus, Fuping Song

**Affiliations:** a State Key Laboratory for Biology of Plant Diseases and Insect Pests, Institute of Plant Protection, Chinese Academy of Agricultural Sciences, Beijing, China; b CAS Key Laboratory of Genome Sciences and Information, Beijing Institute of Genomics, Chinese Academy of Sciences, Beijing, China; c INRA, UMR1319 Micalis, Génétique Microbienne et Environnement, La Minière, Guyancourt, France

## Abstract

We report the draft genome sequence of *Bacillus thuringiensis* strain LM1212, which differentiates into crystal producers or spore formers during the stationary phase. Availability of this genome sequence will facilitate the study of spore formation, crystal formation, cell differentiation, and evolution of *B. thuringiensis*.

## GENOME ANNOUNCEMENT

Here we report the draft genome sequence of *Bacillus thuringiensis* strain LM1212, isolated from the cadaver of an *Oryctes gigas* larva in Madagascar (1). This strain produces a typical crystal inclusion but presents the intriguing ability to differentiate into crystal producers or spore formers during the stationary phase. This characteristic is significantly different from that of all known *B. thuringiensis* strains which produce crystal proteins in the sporulating cells (spore formers).

Total DNA was isolated from *B. thuringiensis* strain LM1212. Genome sequencing was performed with the 454 GS-FLX Titanium (Roche Applied Science) and Illumina Hi-Seq 2000 platforms. A total of 170,744 high-quality Roche 454 reads with an average read length of 400 bp were produced, providing about 11-fold coverage of the genome, while the Illumina reads provided 1,255-fold coverage with 38 million reads of 100 bp (insert size, 3,000 bp). After preprocessing, Roche 454 reads were assembled into contigs with Newbler version 2.6, and then scaffolded with Illumina mate-pair reads using SSPACE (2). The gaps within scaffolds were closed with GapFiller (3). The open reading frames were identified by using Glimmer version 3.02 (4). The tRNAs and rRNAs were predicted using tRNAscan-SE (5) and RNAmmer (6), respectively. The functions of encoding genes were annotated by using the NCBI nr, Swiss-Prot (7), Clusters of Orthologous Groups (COG) (8), KEGG (9), and InterProScan (10) databases.

The draft genome sequence consists of 51 scaffolds and 136 contigs with a GC content of 35.33% and a total length of 6,062,180 bases. A total of 5,286 coding sequences (CDS) and 73 tRNA and 5 rRNA operons were predicted. Approximately 64.16% of all coding sequences (a total of 3,392) were assigned to COG, and 1,814 CDS were annotated into the 166 pathways by using KAAS (11). This strain harbors 7 genes encoding products similar to Cry proteins. Based on the amino acid sequences, five delta-endotoxins in LM1212 (Cry32Va1, Cry74Aa1, Cry45Ba1, Cry41Ca1, and Cry32Mc1) were classified as novel Cry toxins by the Bt Toxin Nomenclature Committee (http://www.lifesci.sussex.ac.uk/home/Neil_Crickmore/Bt/) and four of them (Cry74Aa1, Cry45Ba1, Cry41Ca1, and Cry32Mc1) showed a high degree of similarity to the cancer cell-killing protein parasporins (12).

### Nucleotide sequence accession numbers.

This whole-genome shotgun project has been deposited at DDBJ/EMBL/GenBank under the accession number AYPV00000000. The version described in this paper is version AYPV01000000.

## References

[1] Chaufaux J Marchal M Gilois N Jehanno I Buisson C 1997 Investigation of natural strains of *Bacillus thuringiensis* in different biotopes throughout the world. Can. J. Microbiol. 43:337–343. 10.1139/m97-047

[2] Boetzer M Henkel CV Jansen HJ Butler D Pirovano W 2011 Scaffolding preassembled contigs using SSPACE. Bioinformatics 27:578–579. 10.1093/bioinformatics/btq683 21149342

[3] Boetzer M Pirovano W 2012 Toward almost closed genomes with GapFiller. Genome Biol. 13:R56. 10.1186/gb-2012-13-6-r56 22731987 PMC3446322

[4] Delcher AL Bratke KA Powers EC Salzberg SL 2007 Identifying bacterial genes and endosymbiont DNA with Glimmer. Bioinformatics 23:673–679. 10.1093/bioinformatics/btm009 17237039 PMC2387122

[5] Lowe TM Eddy SR 1997 tRNAscan-SE: a program for improved detection of transfer RNA genes in genomic sequence. Nucleic Acids Res. 25:955–964. 10.1093/nar/25.5.0955 9023104 PMC146525

[6] Lagesen K Hallin P Rødland EA Staerfeldt HH Rognes T Ussery DW 2007 RNAmmer: consistent and rapid annotation of ribosomal RNA genes. Nucleic Acids Res. 35:3100–3108. 10.1093/nar/gkm160 17452365 PMC1888812

[7] Boeckmann B Bairoch A Apweiler R Blatter MC Estreicher A Gasteiger E Martin MJ Michoud K O’Donovan C Phan I Pilbout S Schneider M 2003 The SWISS-PROT protein knowledgebase and its supplement TrEMBL in 2003. Nucleic Acids Res. 31:365–370. 10.1093/nar/gkg095 12520024 PMC165542

[8] Tatusov RL Natale DA Garkavtsev IV Tatusova TA Shankavaram UT Rao BS Kiryutin B Galperin MY Fedorova ND Koonin EV 2001 The COG database: new developments in phylogenetic classification of proteins from complete genomes. Nucleic Acids Res. 29:22–28. 10.1093/nar/29.4.e22 11125040 PMC29819

[9] Kanehisa M Araki M Goto S Hattori M Hirakawa M Itoh M Katayama T Kawashima S Okuda S Tokimatsu T Yamanishi Y 2008 KEGG for linking genomes to life and the environment. Nucleic Acids Res. 36:D480–D484. 10.1093/nar/gkm882 18077471 PMC2238879

[10] Quevillon E Silventoinen V Pillai S Harte N Mulder N Apweiler R Lopez R 2005 InterProScan: protein domains identifier. Nucleic Acids Res. 33:W116–W120. 10.1093/nar/gni118 15980438 PMC1160203

[11] Moriya Y Itoh M Okuda S Yoshizawa AC Kanehisa M 2007 KAAS: an automatic genome annotation and pathway reconstruction server. Nucleic Acids Res. 35:W182–W185. 10.1093/nar/gkm321 17526522 PMC1933193

[12] Ohba M Mizuki E Uemori A 2009 Parasporin, a new anticancer protein group from *Bacillus thuringiensis*. Anticancer Res. 29:427–433 19331182

